# A difficult diagnosis - constrictive pericarditis and its treatment: a case report

**DOI:** 10.1186/1757-1626-2-9105

**Published:** 2009-11-28

**Authors:** Harith A Altemimi, Syed Y Altaf, Rhian K James, Rajah Nata, Eshwar B Kumar, Max Codispoti

**Affiliations:** 1 Department of Cardiology, The Queen Elizabeth Hospital NHS Trust, Kings Lynn, PE30 4ET, UK; 2 University of Cambridge School of Clinical Medicine, Addenbrooke's Biomedical Campus, Cambridge, CB2 0SP, UK; 3 Department of Cardiothoracic Surgery, Papworth Hospital NHS Foundation Trust, Papworth Everard Cambridge CB23 3RE, UK

## Abstract

The diagnosis of constrictive pericarditis requires a high degree of clinical suspicion, for the signs and symptoms of this disease can be falsely attributed to other causes. Herein, we present a case of a 70-year old retired farmer whose symptoms of right heart failure were initially attributed to co-existing pneumonia and pulmonary embolism. He was discharged. Three weeks later he presented with worsening breathlessness and ascites. Echocardiography, computed tomography and cardiac catheterization revealed the diagnosis of constrictive pericarditis. He underwent complete pericardectomy and to date has made a good recovery. This case exemplifies the difficulty in diagnosing this condition, the investigation required, and provides a discussion of the benefit and outcomes of prompt treatment.

## Background

The pericardium is a two-layered sac that encircles the heart consisting of visceral and parietal pericardium. Between these layers is a thin film of liquid, with a total volume of around 50 ml. Constrictive pericarditis is caused by a fibrotic and adherent pericardium restricting the diastolic filling of the heart. The pericardium appears to limit the distension of the myocardium, preventing over-dilatation, and also serves to fix the heart within the mediastinum. It may also act as a barrier to infection spread within the thorax. Complete absence of the pericardium does not appear to adversely affect patients greatly.

## Case Presentation

A 70-year old Caucasian retired farmer presented to the Medical Assessment Unit with a one-week history of shortness of breath, productive cough and tiredness. He had suffered a myocardial infarction 15 years previously and was on insulin for type-two diabetes mellitus. He had no chest pain, leg swelling or fever. On examination, his oxygen saturation was 92% on air with pitting ankle oedema but a normal JVP. Systemic examination was unremarkable. Blood tests showed a normal white cell count but mildly raised CRP and LFTs. Chest x-ray showed right lower lobe consolidation with small bilateral basal effusion and pulmonary congestion. The initial diagnosis of pneumonia was made.

On the third day post admission he suffered increasing shortness of breath, bilateral oedema to the thigh, raised JVP and a distended abdomen. A subsequent CTPA showed bilateral pulmonary emboli and a moderate pericardial effusion. An abdominal ultrasound showed distended hepatic veins in keeping with right ventricular cardiac failure. BNP was only marginally elevated at 125 ng/dL. Through a 4-week admission he was treated for pulmonary embolism, pneumonia and presumed congestive cardiac failure, and was subsequently discharged home with cardiology follow-up.

**Figure 1:**
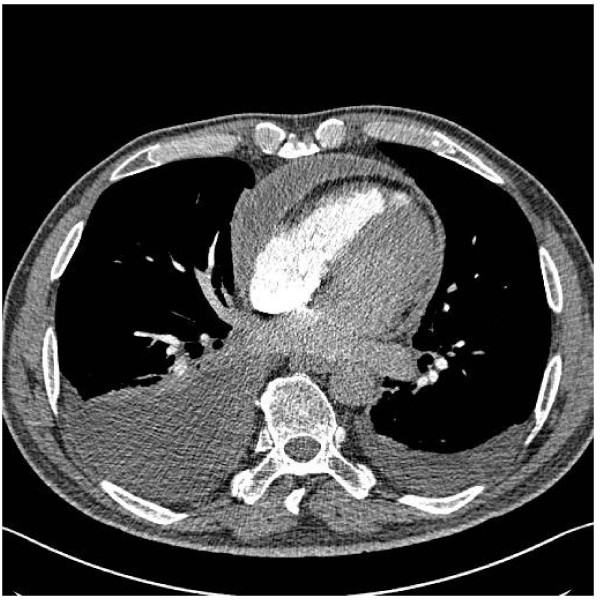
**CT Chest showing thickened pericardium**.

**Figure 2:**
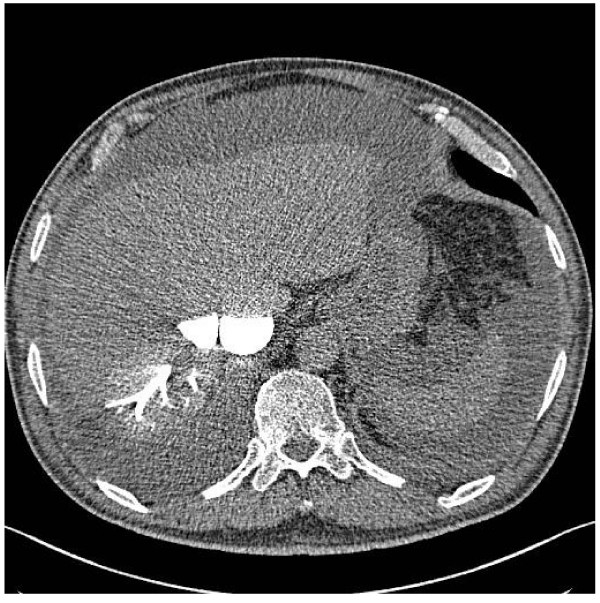
**CT abdomen showing contrast in inferior vena cava and hepatic venous system**.

He re-presented to the same Medical Assessment Unit after just 3 weeks at home with shortness of breath, increasing abdominal distension and bilateral ankle swelling. There was no orthopnoea or paroxysmal nocturnal dyspnoea. On examination he was afebrile there were mild crackles in the bases of both lungs, mild ascites and a raised JVP. Two months after his initial presentation and several weeks into this second admission the suspicion of constrictive pericarditis was raised, which was subsequently confirmed by echocardiography, CT [fig. 1 and fig. 2] and cardiac catheterisation studies.

He was transferred to a tertiary centre where he received a complete pericardectomy and coronary artery bypass grafting. The histology of the resected pericardium and lymph nodes showed no evidence of malignancy or tuberculosis and with no significant medical history the diagnosis of idiopathic constrictive pericarditis was made. He made slow but steady progress post-surgery, and was discharged 18 days later. At 3-months post-op he had no significant symptoms, and was able to walk a couple of miles on the flat.

## Discussion

The diagnosis of constrictive pericarditis is difficult both for its rarity and because it is often obscured by other, more common, diagnoses. The case we present here is of constrictive pericarditis complicated by pneumonia and bilateral pulmonary emboli. The pathophysiology of this disease, in causing a thickened and non-compliant pericardial sack, prevents the diastolic filling phase of the heart. This serves to equalise the pressures in the four chambers during diastole, a subtle sign that may be identified by echocardiography. Functionally, the changes during diastole lead to impaired filling of the heart causing venous back pressure and failure of outflow [1].

In the majority of cases the underlying cause for this change in the pericardium is unknown. Worldwide, tuberculosis and rheumatic heart disease are common causes, and this condition is also known to occur following open heart surgery, radiation, direct trauma, and following viral infection [2]. The signs and symptoms of constrictive pericarditis are variable and often attributed to right heart failure or cirrhosis. The signs relate to the impairment in ventricular filling, causing Kussmaul's sign and pulsus paradoxicus. The systemic venous congestion will cause a raised JVP, hepatic engorgement, pleural effusions, peripheral oedema and ascites. Ascites disproportionate to the degree of hepatic impairment should raise clinical suspicion of constrictive pericarditis [2]. Impairment in cardiac efficiency will lead to fatigue and hypotension. In extreme cases constrictive pericarditis can present in circulatory failure.

Due to the difficulty in the clinical diagnosis, investigations often prove vital in making the diagnosis. Important investigations include echocardiography, CT, MRI and cardiac catheterisation studies. In studies of the diagnostic value of echocardiography, an early diastolic septal bounce was found to be the most specific and consistent sign of constrictive pericarditis, although pericardial adhesions gave the best balance of sensitivity and specificity across the sample [3]. Other diagnostic features on echocardiography include the demonstration of exaggerated interventricular interdependence and a characteristic pattern of ventricular deformation [4].

Cardiac catheterisation studies are necessary for the diagnosis of constrictive pericarditis. Constrictive pericarditis causes early, rapid diastolic filling and equalisation of pressures between the ventricles [5]. One of the greatest challenges of diagnosing constrictive pericarditis is that clinical findings and the results of many investigations are similar to those found in restrictive cardiomyopathy. The predictive accuracy of traditional criteria to distinguish these conditions by cardiac catheterisation is <75% [5]. MRI or CT can be used to demonstrate pericardial thickness of >2 mm, although this is not always consistent.

The diagnosis of constrictive pericarditis is the first and most significant hurdle in preventing further progression of the disease. Despite a two-month delay to diagnosis in this case, our patient benefited from the total pericardectomy which provided symptomatic relief. Partial pericardectomy was found to cause a 4.5 times higher risk of death than total pericardectomy [6] and so total surgical pericardectomy is currently advised for patients with an NYHA class II or III impairment and persisting evidence of constriction [7]. Operative mortality rates range from 11% to 16% and increase with NYHA class [8]. Long-term prognosis is related to the underlying cause. Idiopathic constrictive pericarditis is associated with the best prognosis [9].

## Conclusion

There are many causes of venous congestion, but a rare and commonly missed cause is constrictive pericarditis. Being alert to this as a differential diagnosis is paramount as prompt treatment, including total pericardectomy where indicated, improves quality of life and confers a long-term survival benefit to the patient. Constrictive pericarditis should, therefore, feature on the differential list when a patient presents with signs of right heart failure.

## Abbreviations

JVP: jugular venous pressure; CRP: C-reactive protein; LFTs: liver function tests; CTPA: computed tomography pulmonary angiogram; BNP: brain natriuretic peptide; MRI: magnetic resonance imaging; NYHA: New York Heart Association.

## Competing interests

The authors declare that they have no competing interests.

## Authors' contributions

RJ performed literature search and wrote 1^st ^draft. HA revised the text and was involved in clinical care. SA contributed in literature searching, writing the initial presentation and collated the images. RN and EBK were the lead clinicians. MC was the lead surgeon. All authors read and approved the final manuscript.

## Consent

Written informed consent was obtained from the patient for publication of this case report and accompanying images. A copy of the written consent is available for review by the Editor-in-Chief of this journal.
